# Proceedings of the Michigan State Dental Society

**Published:** 1876-05

**Authors:** 


### Proceedings of Societies.

### PROCEEDINGS OF THE MICHIGAN STATE DENTAL SOCIETY.

The fifteenth annual meeting of the Michigan State, Dental Association commenced its session at Ann Arbor, Wednesday, March 29, 1876. Dr. J. W. Finch, of Adrian presiding. About twenty-five members from different places in the State were present. The first paper was read by Dr. G. R. Thomas, of Detroit, on the "Artificial Separation of the Teeth as a Means of Prevention of Decay." The paper was discussed by Drs. Taft, Robinson, Spelman, Hawxhurst and others.

At the evening session a paper was read by Dr. Hawxhurst, of Battle Creek, on "Physiology," proposing some methods of investigating the progressive development of dental germs during their earlier stages. A discussion followed and was participated in by Drs. Taft, Robinson, Spelman and Jackson.

### THURSDAY MORNING SESSION.

The following amendment to the code of ethics which was presented at the last annual meeting was brought up and adopted, viz:

As the diploma of a legally authorized dental college or university is the formal voucher which furnishes to the community the only presumative evidence of the professional acquirements and abilities of the dentist, so that the possession of such diploma authorizes its holder to affix the title of "doctor" to his name, it is therefore in accordance with etiquette to use this title when addressing such graduate, and equally in bad taste to use it in connection with the name of one who is not a graduate, and as such indiscriminate use of the title tends to depreciate its value it should be carefully avoided.

But as the annals of the profession contain the names of many who, though not having had the advantage of a collegiate education have nevertheless become, through their own exertions and abilities, brilliant scholars and successful, practitioners, therefore, one who enjoys a good moral and professional standing in the community, though not a graduate, should not be excluded from fellowship, but all the honors, respect and consideration usually accorded to professional men, should be freely given him, except the graduation title.

Before its adoption, the amendment was vigorously discussed and was generally favored. It was argued, as a dental society, they ought to take some steps for making the diplomas granted at the Dental School of Michigan University of some value.

Dr. Hawxhurst, of Battle Creek, thought that for the progress and advancement of dental science the resolution ought to be passed. He thought that reform ought to commence with the profession. Adopted.

Dr. Holmes offered a resolution to the effect that a committee of nine be raised to procure a charter for the incorporation of the Michigan Dental Association; also, to memorialize the Legislature for the passage of a law the object of which shall be the protection of the community against quackery in the practice of dentistry in the State of Michigan. Adopted.

A committee of two, consisting of Drs. Holmes and Spelman, was appointed to draft a bill to be presented before the Society for discussion at the afternoon session.

It was decided to hold a special meeting on the second Tuesday in October.

Detroit was selected as the place of meeting in October next.

The following officers were then chosen for the ensuing year:

President—G. R. Thomas, Detroit.

Vice-President—W. D. Tremper, Ypsilanti.

Secretary and Treasurer—E. S. Holmes, Grand Rapids.

Censor—W. H. Jackson, Ann Arbor.

Dr. B. T. Spelman, of Detroit, was elected a member of the University Dental Visiting and Consulting Committee in place of Dr. Finch of Adrian. The other members of the committtee are Drs. Thomas, of Detroit, and Metcalf, of Kalamazoo.

AFTERNOON SESSION.

The resolution adopted at the morning session, appointing nine members as a committee to memorialize the State Legislature was reconsidered and the number was increased to sixteen. The gentlemen selected were as follows: E. S. Holmes, Grand Rapids; G. W. Stone, Ontonagon; A. T. Metcalf, Kalamazoo; G. E. Corbin, St. Johns; E. G. Douglass, Lapeer; J. A. Robinson, Jackson; Thomas Rix, Dowagiac; Wm. M. Andrews, Coldwater; W. R. Cutler, Ionia; J. A. Haris, Pontiac; J. A. Watling, Ypsilanti; J. Douglass, Romeo; J. W. Finch, Adrian; George L. Field, Detroit; D. C. Hauxhurst, Battle Creek; D. W. Smith, Jackson.

A paper was presented by Dr. W. D. Tremper, of Ypsilanti, on "Dental Education." He advocated a more thorough preliminary education before entering the profession, and that every dentist should be a graduate in medicine.

The position taken in the paper was criticised by Dr. Robinson, of Jackson. He claimed that the specialist was the most successful man, and thought that the specialist should be proud of being a specialist.

Dr. Hawxhurst supported the paper, claiming that a thorough medical knowledge is the basis of dentistry.

The paper was discussed by Drs. Spelman and Taft.

Prof. Palmer, having appeared in the meeting, was invited to address the Association. He said he was glad to see that an attempt was being made to raise the standard in the dental profession.

It was decided to hold the next annual meeting at Ann Arbor on the second Tuesday in October, 1877.

